# A pilot study exploring the effect of repetitive transcranial magnetic stimulation (rTMS) treatment on cerebral blood flow and its relation to clinical outcomes in severe enduring anorexia nervosa

**DOI:** 10.1186/s40337-021-00420-w

**Published:** 2021-07-09

**Authors:** Bethan Dalton, Erica Maloney, Samantha J. Rennalls, Savani Bartholdy, Maria Kekic, Jessica McClelland, Iain C. Campbell, Ulrike Schmidt, Owen G. O’Daly

**Affiliations:** 1 Section of Eating Disorders, Department of Psychological Medicine, Institute of Psychiatry, Psychology & Neuroscience, King’s College London, London, UK; 2 Department of Neuroimaging, Institute of Psychiatry, Psychology & Neuroscience, King’s College London, London, UK; 3 South London and Maudsley NHS Foundation Trust, Maudsley Hospital, London, UK

**Keywords:** Anorexia nervosa, Cerebral blood flow, Arterial spin labelling, Functional magnetic resonance imaging, Repetitive transcranial magnetic stimulation, Eating disorders

## Abstract

**Background:**

Repetitive transcranial magnetic stimulation (rTMS) is a novel treatment option for people with severe enduring anorexia nervosa (SE-AN), but associated neurobiological changes are poorly understood. This study investigated the effect of rTMS treatment on regional cerebral blood flow (CBF) and whether any observed changes in CBF are associated with changes in clinical outcomes in people with SE-AN.

**Methods:**

As part of a randomised sham-controlled feasibility trial of 20 sessions of high-frequency rTMS to the left dorsolateral prefrontal cortex, 26 of 34 trial participants completed arterial spin labelling (ASL) functional magnetic resonance imaging (fMRI) to quantify regional and global resting state CBF before (pre-randomisation baseline) and after real or sham treatment (1-month post-randomisation). A group of healthy females (*n* = 30) were recruited for baseline comparison. Clinical outcomes, including BMI, and depression and anxiety symptoms, were assessed at baseline, 1-, 4-, and 18-months post-randomisation.

**Results:**

No group differences in regional CBF were identified between the SE-AN and healthy comparison participants. A significant treatment-by-time interaction in a medial temporal lobe cluster with the maximal peak in the right amygdala was identified, reflecting a greater reduction in amygdala CBF following real rTMS compared to sham. Participants with the greatest rTMS-related reduction in amygdala CBF (i.e., between baseline and 1-month post-randomisation) showed the greatest sustained weight gain at 18-months post-randomisation. Higher baseline CBF in the insula predicted greater weight gain between baseline and 1-month post-randomisation and between baseline and 4-months post-randomisation.

**Conclusions:**

This exploratory pilot study identified rTMS treatment related changes in CBF in adults with SE-AN and these were associated with changes in weight. Our preliminary findings also suggest that CBF (as measured by ASL fMRI) may be a marker of rTMS treatment response in this patient group. Future rTMS studies in AN should employ longitudinal neuroimaging to further explore the neurobiological changes related to rTMS treatment.

**Trial registration:**

ISRCTN14329415, registered 23rd July 2015.

**Supplementary Information:**

The online version contains supplementary material available at 10.1186/s40337-021-00420-w.

## Plain English summary

Repetitive transcranial magnetic stimulation (rTMS) is a novel treatment option for people with severe enduring anorexia nervosa (SE-AN). However, little is known about the neurobiological effects of this treatment. This study explored the effect of rTMS treatment on regional cerebral blood flow (CBF) and whether any observed changes in CBF are associated with changes in clinical outcomes in people with SE-AN. Participants completed arterial spin labelling (ASL) functional magnetic resonance imaging (fMRI) before and after receiving 20 sessions (over 4 weeks) of real or sham rTMS. We found a greater reduction in amygdala CBF following real rTMS compared to sham rTMS. Participants with the greatest rTMS-related reduction in amygdala CBF showed the greatest sustained weight gain at an 18-month follow-up. Higher baseline CBF in the insula predicted greater weight gain during treatment and at a 4-month follow-up. This suggests that CBF (as measured by ASL fMRI) may be a marker of rTMS treatment response in this patient group. Future rTMS studies in AN should use longitudinal neuroimaging to further explore the neurobiological changes related to rTMS treatment.

**Supplementary Information:**

The online version contains supplementary material available at 10.1186/s40337-021-00420-w.

## Introduction

Anorexia nervosa (AN) is a disabling and deadly disorder associated with physical and psychological morbidity and impaired quality of life [1]. Psychological therapy is often the treatment of choice for adults with AN; however, with these best available treatments, recovery rates are 13–50% at 1–2 years follow-up [2]. Approximately 20–30% of AN patients develop a chronic form of the disorder [3, 4], termed severe enduring AN (SE-AN, [5]), and after 3–5 years of illness, outcomes become significantly poorer [1, 6]. Thus, treatment innovations are needed. Research into the neural underpinnings of AN have provided a rationale for the investigation of targeted brain-directed interventions [7, 8]. Repetitive transcranial magnetic stimulation (rTMS) has shown potential as a treatment for SE-AN (e.g., [9, 10]). rTMS is a non-invasive form of brain stimulation that can promote (using high-frequency) or inhibit (using low-frequency) cortical activity in a target brain area and produces effects that exceed the duration of the initial stimulation period [11]. As rTMS appears to increase neuroplasticity [12], it is thought to be of value in chronic illnesses, such as SE-AN, which are likely to be associated with changes in neurocircuitry. However, despite the initial promise of this brain-directed intervention in this patient group, neurobiological changes associated with rTMS, and their relationship to clinical outcomes, have only been explored in a few studies of people with eating disorders (EDs) [13, 14]. Such studies will be important for developing an understanding of the mechanisms and predictors of response underlying rTMS treatment in this patient group.

We recently completed a randomised controlled feasibility trial of 20 sessions of real versus sham high-frequency (10 Hz) rTMS to the left dorsolateral prefrontal cortex (DLPFC) in 34 women with SE-AN (the TIARA trial, [15]). Outcomes provided preliminary evidence for the therapeutic potential of rTMS in SE-AN [16, 17]. Specifically, real rTMS, relative to sham treatment, showed moderate to large effects on anxiety and mood outcomes between baseline (pre-randomisation) and 4-months post-randomisation. The positive effects on mood were maintained at an open 18-month post-randomisation follow-up. These persistent improvements in affective symptoms replicated the findings from a small case series that explored rTMS treatment in SE-AN [18]. In the TIARA trial, there also appeared to be an rTMS effect on body mass index (BMI) change between baseline and 18-months post-randomisation, with greater weight gain in those originally allocated to the real, compared to sham, rTMS group. While neurobiological changes associated with rTMS have yet to be explored in SE-AN, they have been investigated in two small studies of people with EDs characterised by binge-eating and purging. In these, rTMS was associated with reductions in haemoglobin concentrations (assessed using near-infrared spectroscopy) in the rTMS-target brain area [14] and changes in functional connectivity [13]. The latter study also explored predictors of rTMS response and found that participants who responded to rTMS, compared to non-responders, showed baseline hypoconnectivity from the DMPFC to other cortical and subcortical regions, including those involved in emotion generation and regulation (e.g., insula) and significantly greater rTMS-related increases in frontostriatal and fronto-insular connectivity [13]. This highlights the potential of neuroimaging as a tool to optimise rTMS treatment.

Cerebral blood flow (CBF), an indirect marker of neuronal activity, has been explored as a neural correlate of the effects of rTMS in neuroimaging studies in healthy individuals (e.g., [19, 20]) and people with depression (e.g., [21]). This research has reported that CBF is altered during and following rTMS treatment. High-frequency rTMS to the left DLPFC has been associated with increased CBF in this region (e.g., [21–24]), and rTMS targeted at frontal regions has also been reported to lead to more widespread changes in CBF. For example, increases in CBF following rTMS targeted at the prefrontal cortex have been observed in the hippocampus, left amygdala, and bilateral insula [20, 24]. However, the direction of these more distal effects of DLPFC-targeted rTMS on CBF varies, possibly owing to differences in sample characteristics and data pre-processing. Preliminary research has also shown that CBF is associated with rTMS treatment efficacy, though findings are mixed (e.g., [25–27]). For example, baseline CBF in the insular cortex has been negatively [25] and positively [26] correlated with early response to rTMS treatment in patients with depression. Taken together, CBF may be a valuable neural correlate and marker of rTMS treatment effects.

Imaging studies have shown that CBF is altered in people currently unwell with AN (compared to healthy individuals) in posterior cingulate gyrus and temporal areas [28, 29]. However, some studies have identified increases (e.g., [29]) and others identified reductions (e.g., [30]) in CBF in these areas, compared to healthy individuals. Preliminary research also suggests that CBF changes in response to weight gain and recovery in AN [28], e.g., following weight gain, CBF has been reported to increase in the right DLPFC, posterior and anterior cingulate cortex and the parietal lobe, and to decrease in the right amygdala [31–33]. Given these findings in people with AN, along with the reported effects of rTMS on CBF, an exploration of the effect of rTMS on CBF in people with SE-AN and whether this is associated with clinical outcomes is warranted.

The DLPFC plays a key role in self-regulatory control mechanisms and has been implicated in AN, which has been described as a disorder of excessive self-control [34]. The DLPFC has been associated with cognitive control processes in AN, including inhibitory control, food choice, emotion regulation, reward processing, among other processes [7, 35, 36]. The DLPFC is heavily interconnected with limbic regions, including the amygdala and insula, and dysregulation in this fronto-limbic circuit (including the anterior cingulate cortex) has been associated with behavioural and emotional regulation and responses in AN [37]. Taken together, rTMS targeting the left DLPFC may therefore alter the top-down control of the DLPFC to fronto-limbic regions associated with maladaptive emotion regulation strategies (e.g., dietary restraint), and subsequently improve ED and affective symptoms.

In our TIARA trial, before and after completion of 4 weeks of rTMS treatment, we performed at rest arterial spin labelling (ASL) functional magnetic resonance imaging (fMRI), a non-invasive method that provides a quantitative measure of CBF. This pilot analysis aimed to investigate (a) the effect of rTMS treatment on regional CBF at post-treatment (1-month post-randomisation); (b) whether any rTMS-related changes in CBF were associated with change in clinical outcomes at post-treatment (1-month post-randomisation) and follow-up (4- and 18-months post-randomisation); and (c) whether baseline CBF could predict clinical outcomes in the shorter- and longer-term. A secondary aim was to investigate group differences in CBF between SE-AN and healthy comparison participants. Analyses were performed from an exploratory whole brain perspective and also focussed on four regions of interest (bilateral DLPFC, amygdalae, anterior cingulate cortices, insular cortices).

## Methods

This study used data collected as part of a double-blind, parallel group, two-arm, sham-controlled randomised feasibility trial (Trial Registration: ISRCTN14329415, registered 23rd July 2015, https://www.isrctn.com/ISRCTN14329415). Methodological details have been described in Bartholdy, McClelland et al. [15] and clinical outcomes reported in Dalton, Bartholdy, McClelland, et al. [16] and Dalton et al. [17]. Ethical approval was obtained from London - City Road & Hampstead Research Ethics Committee (Ref: 15/LO/0196).

### Participants

Thirty-four female adults with a current Diagnostic and Statistical Manual of Mental Disorders (DSM)-5 [38] diagnosis of AN and a BMI > 14 kg/m^2^ were recruited. Participants had a severe enduring form of AN (defined as illness duration ≥3 years and completion of at least one previous specialist treatment for their ED). Participants were recruited from a specialist Eating Disorder Service in London, through online advertisements (e.g., Beat, the UK national ED charity) and social media, and via participation in previous studies.

Healthy comparison (HC) women (*n* = 30; ≥18 years), with a BMI in the healthy range (20–25 kg/m^2^), were recruited via online and poster advertisements at King’s College London (KCL) to provide a comparison group. Exclusion criteria were current/past psychiatric illness or a family history of an ED. HCs completed the baseline assessment only.

Exclusion criteria for all participants included left-handedness, MRI contraindications (e.g., metallic implants), current and/or history of neurological illness, and additionally for SE-AN participants, TMS contraindications (e.g., seizures). Participants completed a telephone screening to confirm their eligibility. This included the TMS Adult Safety Screen for SE-AN participants only; an MRI safety screen questionnaire developed at KCL; the Eating Disorder Diagnostic Scale [39] to assess the presence/absence of ED symptoms in the SE-AN and HC groups, respectively; the researcher version of the Structured Clinical Interview for DSM-IV Axis I Disorders Screening Module [40] to confirm the absence of any psychiatric disorders in the HC participants; and a short inclusion/exclusion screen specific to this study. Written informed consent was obtained from all participants.

### Procedure

All participants completed a baseline assessment consisting of weight and height measurements, a questionnaire pack, neuropsychological computer tasks, and a one-hour MRI scan. Following the baseline assessment, SE-AN participants were randomly allocated to receive 20 sessions (every weekday for 4 weeks) of real or sham neuro-navigated rTMS targeting the left DLPFC (Talairach co-ordinates x = − 45, y = 45, z = 30, as used by [41]). The left DLPFC was selected as the target brain area for rTMS in this trial for several reasons: (a) the DLPFC is involved in a number of cognitive processes that have been implicated in AN e.g., emotion regulation, self-control (as described in [7, 42]); (b) high-frequency rTMS to the left DLPFC has shown efficacy and acceptability in the treatment of related psychiatric disorders, including depression [43]; and (c) for practical accessibility reasons. rTMS was administered by trained researchers using the Magstim Rapid device (Magstim®, Whitland, Wales, UK) and Magstim d70-mm-air-cooled real/sham figure-of-eight coil. Participants received 20 min of high-frequency (10 Hz) rTMS consisting of 20 five-second trains with 55-s intervals at 110% of participants’ resting motor threshold. Participants allocated to the sham group received rTMS at the same parameters using a sham coil. These stimulation parameters were selected based on protocols used in other ED related rTMS research by our group [44, 45] and were in accordance with safety and application guidelines for rTMS [11].

**Fig. 1:**
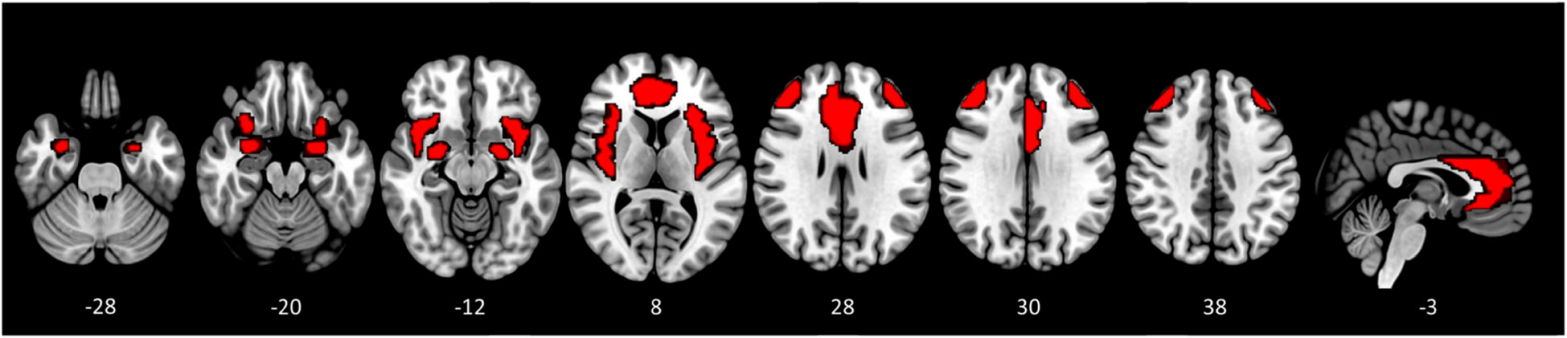
Brain mask used for all hypothesis-led analyses, including all voxels for the amygdala, insula and anterior cingulate cortices bilaterally and the rTMS-targeted region of the DLPFC and its contralateral mirror

SE-AN participants repeated the baseline assessment within 1 week of completing rTMS treatment (post-treatment; 1-month post-randomisation) and at a short-term follow-up (4-months post-randomisation; without the neuroimaging component) prior to unblinding. After this, sham treatment completers were given the opportunity to receive real rTMS treatment (identical protocol and rTMS parameters to those used in the main trial) if they remained eligible. Participant flow through the trial is shown in the Supporting Information (S1: Figure 1). At an open follow-up (18-months post-randomisation), SE-AN participants completed a short questionnaire pack and reported their current BMI.

### Clinical outcome measures

BMI and depression and anxiety were selected as clinical outcome measures of interest in the present analyses due to the observed rTMS treatment effects on these variables in the trial. In relation to BMI, we reported a medium between-group effect size for BMI change from baseline to 18-months post-randomisation, favouring those originally allocated to real rTMS over sham, and also a greater rate of weight recovery (BMI ≥18.5 kg/m^2^) in the real compared to original sham rTMS group [46]. In relation to depression and anxiety, we reported moderate to large effect sizes for changes in these outcomes from baseline to 4-months post-randomisation and it was concluded that general psychopathology should serve as a primary outcome for future rTMS trials in AN [16]. We also identified somewhat higher depression scores in the trial, compared with other treatment trials in AN [47, 48]. More broadly, both depression and anxiety are highly comorbid with AN [49] and comorbid depression has been associated with poor quality of life in SE-AN [50].

BMI was calculated as weight (in kilograms) divided by height (in metres) squared. Depression and anxiety were measured using the relevant subscales of the Depression, Anxiety and Stress Scales – Version 21 (DASS-21 [51]). The DASS-21 is widely used and has been reported to have acceptable to excellent internal consistency and concurrent and divergent validity in clinical and non-clinical samples [52]. Data on other clinical outcomes are presented elsewhere [16, 17].

### Arterial spin labelling functional magnetic resonance imaging

Scanning procedures were carried out at the Centre for Neuroimaging Sciences, Institute of Psychiatry, Psychology & Neuroscience, KCL, using a General Electric MR750 3.0 T scanner.

Pseudo-continuous arterial spin labelling (pCASL) was used to non-invasively obtain a quantitative measure of CBF. Blood water protons are magnetically labelled as the blood flows through the carotid artery to the brain. This is achieved by applying a long radio-frequency pulse to invert the magnetization of the arterial blood water which acts as a diffusible tracer or contrast agent [53–55]. Two whole volume images are acquired: one with and one without the labelled arterial blood. The pairwise subtraction of these images indicate the volume of blood perfused into the cerebral tissue during the time between labelling and image acquisition.

CBF at rest was measured in each participant using pseudo continuous flow-driven adiabatic inversion scheme [56]. Fifty-six slices were acquired with a thickness of 3 mm and a slice gap of 3 mm using the following parameters: 240 mm FoV, 512 × 8 acquisition matrix, TR/TE of 5135 ms/11.088 ms and flip angle of 111O. The inversion time (TI) between the pulse labelling and the beginning of the readout was 2025 ms for all subjects. CBF maps were calculated in real time on the MRI scanner. A proton density scan was acquired employing the same parameters and was used as a reference image when calibrating the CBF map [57]. A structural T1-weighted (MP-RAGE) scan was performed and used as a co-registration of each individual’s imaging data into a common space for processing and analysis, using the following sequence parameters: FoV 270 mm; flip angle 11^o^; matrix size 256 × 256; 196 slices with a slice thickness and slice gap of 1.2 mm; TR = 7.312; TE = 3.016; voxel size 1.1 × 1.1 × 1.2. The MP-RAGE scan was also used in the localisation of the left DLPFC for rTMS treatment.

### Image processing and analysis

CBF was processed and analysed voxel-by-voxel using Statistical Parametric Mapping software version 12 (SPM12, Wellcome Trust Centre for Neuroimaging, London, UK) running under Matlab 8.2.0 (MathWorks Inc., Natick, MA, USA). Structural images were manually reoriented so that the image origin was set at the anterior commissure (AC), providing better normalisation to the MNI (Montreal Neurological Institute) space and controlling for unintentional subject movement. Structural scans of all participants were segmented into grey matter (GM), white matter (WM) and cerebrospinal fluid (CSF) probability maps. Based on GM segmentations, a population-based template was created using Diffeomorphic Anatomical Registration using Exponentiated Lie Algebra (DARTEL) to improve registration of structural images. Participants with AN have been found to have reduced brain size and structural brain changes when compared with age-matched healthy controls [58–60] and based on this, it was decided a study-specific template would be created. DARTEL allows for enhanced accuracy of inter-subject alignment by modelling the shape of each brain using three parameters for each voxel [61].

Rigid-body parameters to align the CBF images to the MP-RAGE image were calculated via co-registration of the proton density image, which is in perfect register with the CBF map, to the structural T1-weighted image. Parameters were then applied to the CBF maps to bring them into register with the MP-RAGE image. Following this, the flow fields created by the DARTEL template were applied to the CBF maps and from there, the images were normalized to MNI (standard) space. The CBF scans were then smoothed using an 8 mm full width at half maximum (FWHM) Gaussian Kernel.

To determine if any regional difference (between groups) or changes (over time) in CBF simply reflect changes in underlying tissue volume, a voxel-based morphometry analysis was carried out. The data were pre-processed in accordance with the published methodology [62]. In brief, T1-weighted structural images underwent unified segmentation, and the resultant GM and WM segmentations were up-sampled (to 1.5 mm × 1.5 mm × 1.5 mm) and used to generate the study-specific DARTEL template mentioned earlier. The tissue segmentations were normalized to standard space via the intermediate template and again smoothed by an 8 mm FWHM smoothing kernel. Total intracranial volume (TIV) was calculated using an in-house script.

#### Exploratory whole-brain analyses

For GM, WM and CBF data, whole brain analyses were implemented in SPM. To test for baseline group-differences in GM, WM and CBF between the HC and SE-AN group, independent sample t-tests were used. To test for rTMS-related effects in the SE-AN group, a flexible-factorial ANOVA was implemented. This permits modelling with appropriate control for between-subjects’ variance when testing for a treatment-by-time interaction. Finally, to determine whether baseline CBF might be a predictive marker of subsequent rTMS treatment response, we conducted regression analyses to explore the relationship between baseline CBF and the change (either from pre-treatment baseline to 1-month post-randomisation or from pre-treatment baseline to 4-months post-randomisation) in those participants who received real rTMS. These regression analyses focussed on three parameters: BMI, and anxiety and depression (as measured by the DASS-21). Given evidence of further weight gain at the 18-month post-randomisation follow-up [17], we also tested whether baseline and treatment-related change in regional CBF predicted sustained change in these variables.

For the independent samples t-test, age and BMI were included as covariates of no interest. For the AN-specific ANCOVA and regression analyses, AN type (AN-restricting [AN-R] / AN-binge-eating/purging [AN-BP]) and the number of hospitalisations (as a proxy measure for disease severity/chronicity) were included as nuisance covariates. For all VBM analyses, the TIV was included as an additional covariate of no interest, whereas for the CBF analysis, the global GM CBF was calculated (using SPM global calculation) and included as a nuisance covariate using the ANCOVA option under global normalization.

For all exploratory whole-brain analysis, results were considered significant if they survived whole-brain familywise error correction on the basis of cluster extent with a critical corrected alpha of 0.05 (i.e., *p*FWE < 0.05).

#### Regions of interest (ROI) definition and analysis

Regions of interest (ROI) analysis was conducted for the primary contrast of interest. All ROIs were defined using the WFUPickAtlas toolbox (SCR_007378; RRID: nif-0000-00358) [63] available in SPM. Selection of ROIs was based on *a priori* assumptions and literature detailing the involvement of these structures in prominent behaviours and characteristics associated with AN [7, 36]. All structures were defined using the Automated Anatomical Labelling (AAL) library [64]. The resultant bilateral masks for the amygdalae, anterior cingulate cortices, and insular cortices were combined with bilateral DLPFC ROIs based on the site of rTMS delivery. Specifically, a 15 mm sphere was centred extra-cerebrally on the Talairach coordinate targeted during rTMS ([x = − 45 y = 45 z = 30]) and its mirror contralaterally. The intersection between these spheres and the GM mask (thresholded at 20% probability of mask) served as the DLPFC mask. The final combined mask (see Fig. 1) was used to spatially constrain familywise error correction for hypothesis-led analyses (i.e., small volume correction) with significance defined as a corrected *p*-value of *p*FWE< 0.05 on the basis of response amplitude.

## Results

### Cross-sectional comparison of baseline cerebral blood flow

At baseline, MRI data were available for 30 HC and 26 SE-AN participants (see Table 1 for demographics). Data from eight SE-AN participants were excluded due to incomplete scans and datasets. Specifically, *n* = 3 SE-AN participants only completed the scanning procedure required for neuronavigation in the rTMS treatment for safety reasons, *n* = 1 did not complete the ASL scan due to technical issues, and the remaining *n* = 4 were excluded due to data quality issues.

**Table 1:** Baseline demographics and clinical characteristics for the healthy comparison and severe enduring anorexia nervosa participants

	HC (*n* = 30)	SE-AN (*n* = 26)
Age [years] (mean ± SD)	25.57 ± 4.02	27.62 ± 9.11
Illness duration [years] (mean ± SD)		13.02 ± 9.81
AN-R/AN-BP [n]		16 / 10
EDE-Q Global (mean ± SD)	0.42 ± 0.43	4.17 ± 1.13
BMI [kg/m^2^] (mean ± SD)	21.87 ± 1.55	16.06 ± 1.40
DASS-21 Depression (mean ± SD)	1.93 ± 3.70	26.85 ± 10.08
DASS-21 Anxiety (mean ± SD)	1.53 ± 1.87	15.69 ± 10.24

*Abbreviations*: *HC* healthy comparison, *SE-AN* severe enduring anorexia nervosa, *SD* standard deviation, *AN-R* anorexia nervosa restricting type, *AN-BP* anorexia nervosa binge-eating/purging type, *EDE-Q* Eating Disorder Examination Questionnaire, *BMI* body mass index, *DASS-21* Depression, Anxiety and Stress Scale –Version 21

#### Hypothesis-led ROI analysis

The hypothesis-led analysis of baseline data found no differences in regional CBF between the HC participants and those with SE-AN (all familywise error corrected *p*-values (*p*FWE > 0.409).

#### Exploratory whole-brain analysis

We found no evidence for significant regional differences in baseline CBF between HC participants and those with SE-AN.

#### Confirmatory structural analyses

Neither hypothesis-led nor exploratory voxel-brain analyses revealed any regional difference between the HC and SE-AN groups either in GM (all *p*-values > 0.52) or WM (all *p*-values > 0.396). However, using the mask of the bilateral amygdala only, we found evidence of less WM volume in a region of the right amygdala in the SE-AN group compared to the HC group (*p*FWE_SVC_ = 0.044, Z = 3.55, [32–4 -28]; see Supporting Information S2: Table 1 for effect sizes).

### Effect of rTMS on cerebral blood flow and the association with clinical outcomes

Twenty-four SE-AN participants (*n* = 14 real rTMS, *n* = 10 sham rTMS) had complete MRI scans and datasets at both baseline and post-treatment (1-month post-randomisation). Table 2 shows the baseline demographics and clinical characteristics for these SE-AN participants by rTMS treatment arm. Of those who received real rTMS, all (*n* = 14) also had clinical data available at 4-months post-randomisation and *n* = 11 (except for BMI where *n* = 10) at 18-months post-randomisation for the exploratory analyses on the association between CBF and clinical outcomes.

**Table 2:** Baseline demographics and clinical characteristics for severe enduring anorexia nervosa participants included in the present analyses, presented for each treatment group (real and sham) separately

	Real rTMS (***n*** = 14)	Sham rTMS (***n*** = 10)
Age [years] (mean ± SD)	28.79 ± 10.17	26.20 ± 8.19
Illness duration [years] (mean ± SD)	13.96 ± 11.58	11.70 ± 7.72
AN-R/AN-BP [n]	8 / 6	6 / 4
EDE-Q Global (mean ± SD)	3.95 ± 1.38	4.38 ± 0.76
BMI [kg/m^2^] (mean ± SD)	15.93 ± 1.67	16.47 ± 0.73
DASS-21 Depression (mean ± SD)	26.43 ± 10.14	26.40 ± 11.23
DASS-21 Anxiety (mean ± SD)	14.86 ± 8.80	16.40 ± 13.16

*Abbreviations*: *rTMS* repetitive transcranial magnetic stimulation, *SD* standard deviation, *AN-R* anorexia nervosa restricting type, *AN-BP* anorexia nervosa binge-eating/purging type, *EDE-Q* Eating Disorder Examination Questionnaire, *BMI* body mass index, *DASS-21* Depression, Anxiety and Stress Scale –Version 21

For effect sizes and post-hoc power analyses for the following significant results, see the Supporting Information S2: Table 1.

#### rTMS effects on regional cerebral blood flow

##### Hypothesis-led ROI analysis

**Fig. 2:**
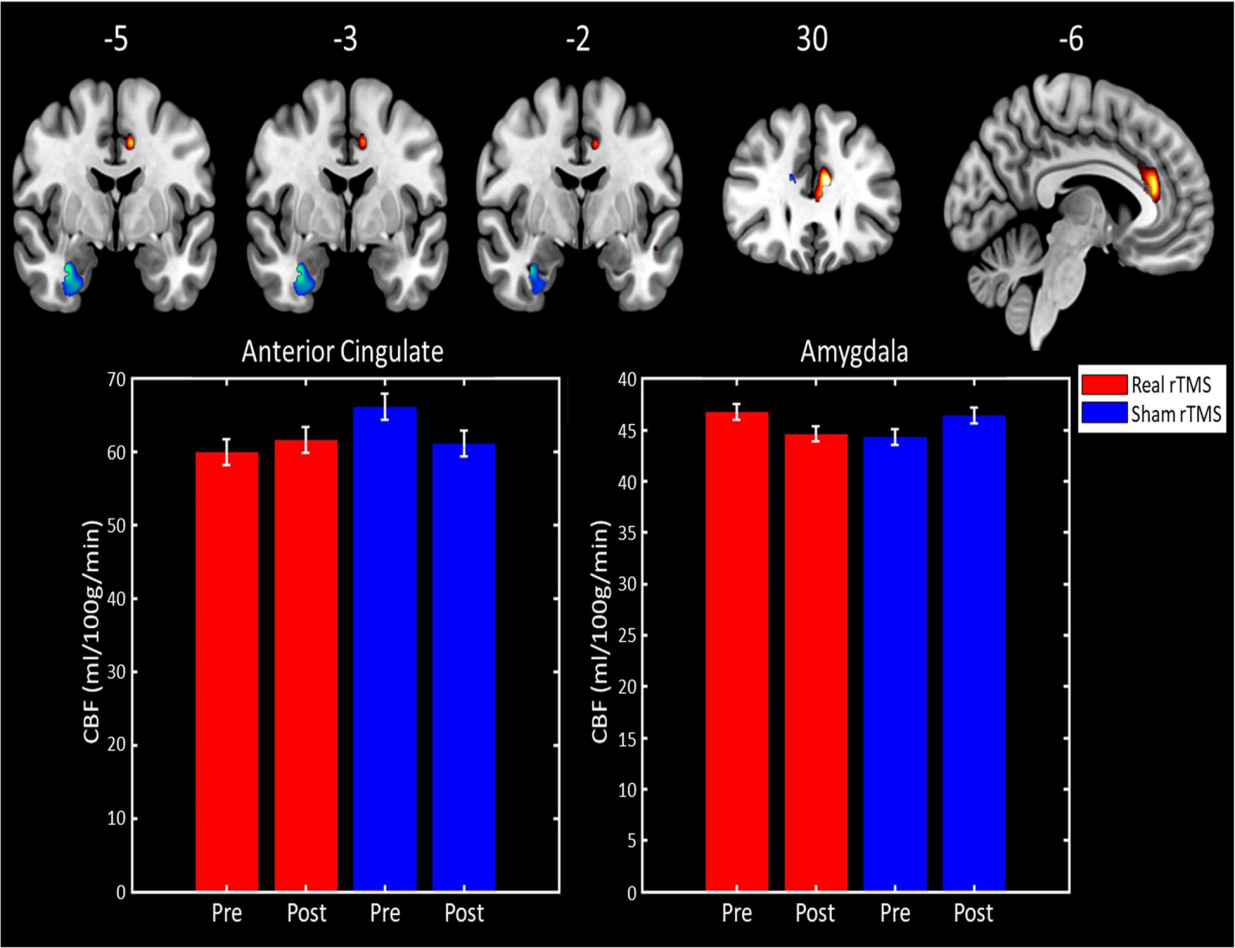
*Top:* Brain regions where regional CBF demonstrated a treatment-by-time interaction. Regions in blue have an interaction driven by treatment-related decreases in CBF following real rTMS, whereas those in yellow/red are driven by CBF reduction in the sham rTMS group. Brain images shown at an uncorrected threshold of *p* < 0.005, slice numbers are in MNI space. *Bottom*: Bar graphs showing the cluster mean regional CBF values for the anterior cingulate cluster (left) and right amygdala (right) for the real rTMS (in red) and sham rTMS (in blue) groups

Testing for changes in regional CBF from baseline to the post-treatment scan (i.e., 1 month later) revealed a significant treatment-by-time interaction in a medial temporal lobe cluster with the maximal peak in the right amygdala (*p*FWE_SVC_ = 0.014, Z-score = 4.26, peak at [33–3 -26]). This reflected a significantly greater reduction in amygdala CBF following real rTMS compared to sham (see Fig. 2). The same analysis also revealed an interaction in the anterior cingulate cortex, which did not survive familywise error correction (*p*FWE_SVC_ = 0.074, Z = 3.78, [− 9 32 20]).

##### Exploratory whole-brain analysis

No regional treatment-by-time interactions were observed in CBF. All whole-brain CBF analyses were constrained by a GM mask, generated by thresholding SPMs GM tissue probability map to include only voxels with at least a 20% probability of being classified as GM.

##### Confirmatory structural analyses

We found no evidence for treatment-by-time interaction in GM (*p*FWE_SVC_ > 0.762) or WM (*p*FWE_SVC_ > 0.322; *p*FWE_SVC_ = 0.158 using amygdala mask only) that survived familywise error correction.

#### Association between rTMS-related changes in cerebral blood flow and change in clinical outcomes

##### Hypothesis-led ROI analysis

We first explored the relationship between changes in CBF (baseline to post-treatment scan at 1-month post-randomisation) and change in our three clinical outcomes of interest (BMI, anxiety and depression) both from baseline to 1-month post-randomisation and from baseline to the 4-month post-randomisation follow-up. No significant associations using a bilateral mask in three of our ROIs (anterior cingulate cortex, amygdala and insula) (all *p*FWE_SVC_ > 0.31) were identified. However, when using a bilateral amygdala mask only, a regional change in left amygdala CBF was weakly associated with the change in anxiety over the treatment window (baseline to 1-month post-randomisation) (*p*FWE_SVC_ = 0.051, Z = 3, [− 21–9 -10]).

**Fig. 3:**
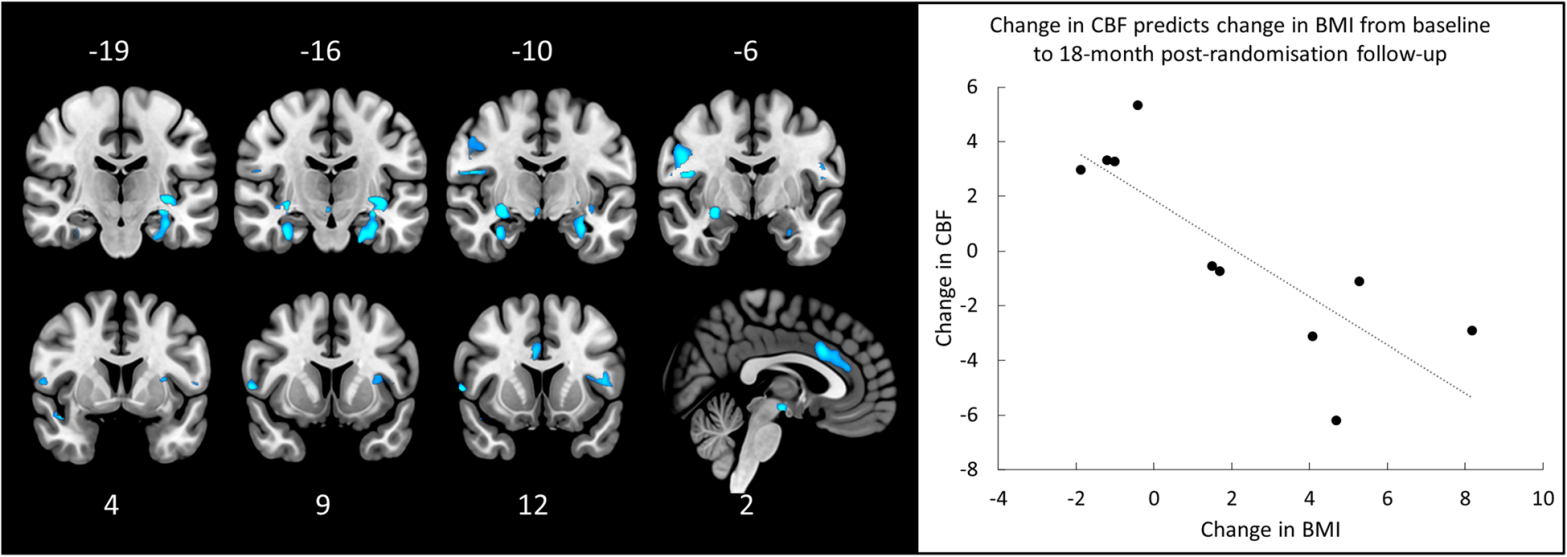
*Left*: Brain regions where rTMS-related change in CBF significantly predicts sustained increases in BMI at the 18-month post-randomisation follow-up. *Right*: Scatter plot, with trend-line added, illustrating the association between change in CBF (baseline to post-treatment [1-month post-randomisation]) and change in BMI (baseline to 18-months post-randomisation), while controlling for AN type and chronicity i.e., reduction in CBF during treatment is associated with long-term increases in BMI. Brain images shown at an uncorrected threshold of *p* < 0.005, slice numbers are in MNI space

Based on findings from the 18-month post-randomisation follow-up [17], we explored whether rTMS-related change in CBF (post-treatment [1-month post-randomisation] minus baseline) predicted change in BMI, anxiety and depression over this longer time-period in a subset (*n* = 11, except for BMI where *n* = 10) of participants who received real rTMS and responded to this follow-up. The change in BMI between baseline and 18-months post-randomisation was significantly negatively correlated with rTMS-related change in amygdala regional CBF (*p*FWE_SVC_ = 0.033, Z = 4.56, [28–9 -12]; see Fig. 3) i.e., those with the greatest rTMS-related reduction in amygdala regional CBF showed the greatest sustained weight gain measured at 18-months post-randomisation. However, the association between BMI change and change in regional CBF in the insular cortex did not survive FWE correction (*p*FWE_SVC_ = 0.096, Z = 4.07, [− 36–16 -3]). To confirm the regional specificity, we tested each region separately using separate bilateral ROIs for the amygdala (*p*FWE_SVC_ = 0.003) and the insular cortex (*p*FWE_SVC_ = 0.048). Changes in CBF were not associated with changes in either anxiety or depression.

#### Association between baseline CBF and short- and longer-term clinical outcomes

##### Hypothesis-led ROI analysis

**Fig. 4:**
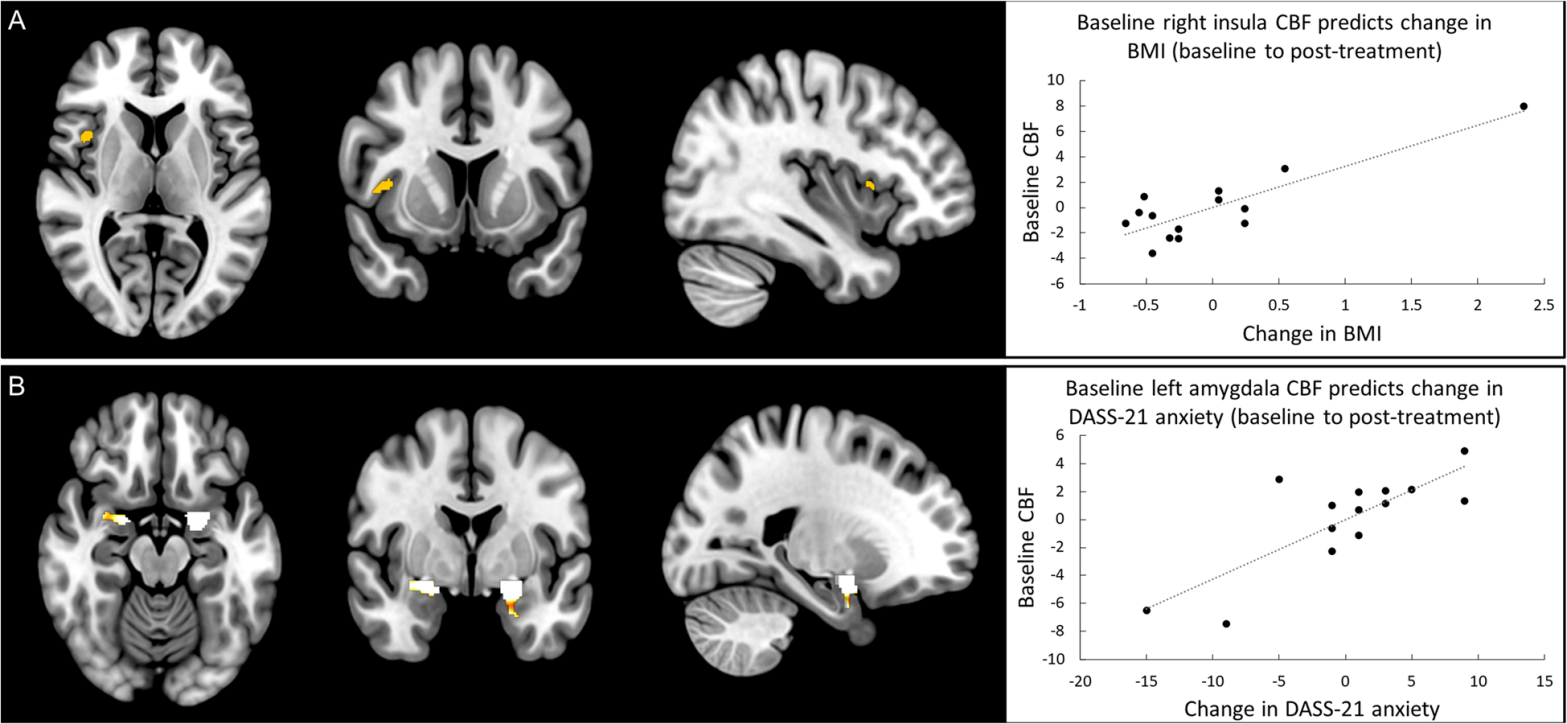
**A** *Left*: Brain regions where baseline CBF significantly predicts change in BMI between baseline and post-treatment (1-month post-randomisation). *Right*: Scatter plot, with trend-line added, illustrating the association between baseline CBF and change in BMI, while controlling for AN type and chronicity i.e., lower baseline right insula CBF is associated with increases in BMI. **B** *Left*: Brain regions where baseline CBF significantly predicts change in DASS-21 anxiety between baseline and post-treatment (1-month post-randomisation). *Right*: Scatter plot, with trend-line added, illustrating the association between baseline CBF and change in anxiety, while controlling for AN type and chronicity i.e., lower baseline left amygdala CBF is associated with a reduction in anxiety. Brain images shown at an uncorrected threshold of *p* < 0.005, slice numbers are in MNI space

Regression analyses demonstrated that baseline CBF in the insula positively predicted the change in BMI seen over the one-month treatment period (i.e., 1-month post-randomisation minus baseline; *p*FWE_SVC_ = 0.019, z = 4.51, [40 10 6]; see Fig. 4A) and also the difference from baseline to the 4-month post-randomisation follow-up (i.e., follow-up minus baseline; *p*FWE_SVC_ = 0.025, Z = 4.43, [39 9 8]).

With respect to anxiety, the change from baseline to 1-month post-randomisation was positively correlated with the baseline CBF in the left (*p*FWE_SVC_ = 0.008, Z = 4.79, [− 20–8 -12]; see Fig. 4B), but not the right (*p*FWE_SVC_ < 0.323, Z = 3.49, [20–2 -14]), amygdalae, unless a bilateral amygdala mask was used (*p*FWE_SVC_ = 0.034). However, baseline CBF in the left amygdala was no longer significantly associated with anxiety when the change from baseline to the 4-month post-randomisation assessment was examined (*p*FWE_SVC_ = 0.811; *p*FWE_SVC_ = 0.149 using bilateral amygdala mask only). No results were significant for depression (all *p*-values> 0.51).

We also explored whether baseline CBF predicted change in BMI, anxiety and depression from baseline to 18-months post-randomisation (*n* = 11, except for BMI where *n* = 10). We found no significant relationship between baseline CBF and changes in the outcomes of interest at 18-month post-randomisation (all *p*FWE > 0.637).

##### Exploratory whole-brain analysis

We found no additional associations between baseline CBF and change in any of the three measures of interest in those that received real rTMS.

## Discussion

In this exploratory study, we examined changes in CBF, quantified using pseudo-continuous ASL fMRI, following high-frequency rTMS to the left DLPFC in people with SE-AN. We also considered how any CBF changes were associated with changes in clinical outcomes and explored whether baseline CBF may be a potential marker of rTMS treatment response. As part of this, we investigated differences in CBF between SE-AN and HC participants, identifying no group differences. This is consistent with findings from two studies that quantified CBF using positron emission topography (PET) [65, 66].

Unlike previous rTMS studies, we found no evidence of increased CBF at the stimulation site following rTMS treatment [21–24]. In studies of CBF in depression [67], the DLPFC has been implicated in rumination, as opposed to a distracted condition, and such resting ruminative activity may explain the evidence for TMS-related modulation of “resting” CBF in that region. For the SE-AN group in our study, in the absence of symptom-provoking stimuli, the DLPFC may not be recruited in a sufficiently sustained manner to change the mean CBF over the scan window (ASL produces an image of average CBF over approximately five-minutes). We did, however, find that amygdala CBF significantly reduced following real rTMS, compared to sham treatment. Although the SE-AN group had less WM in a similar region of the right amygdala, compared to HC participants at baseline, we found no evidence for significant treatment-related structural changes and no association between illness duration and the observed amygdala effects (see Supporting Information S3 and S3: Figure 2). Reasons for reduced perfusion in the right amygdala following real rTMS are unclear, particularly as high-frequency (i.e., excitatory) rTMS was applied to the left DLPFC. Although we did not identify any rTMS related changes in CBF in the DLPFC, this is a densely connected neural hub and so, stimulation to this area could cause widespread changes across brain networks. Indeed, it has been proposed that the DLPFC has indirect projections to the amygdala (e.g., [68, 69]) and rTMS studies targeting the DLPFC in the treatment of depression have reported distal effects on CBF in the amygdala [70]. However, the lateralisation, contralateral effects, and direction (i.e., reduction) of our findings is somewhat contradictory to previous findings in a small study of people with depression i.e., high-frequency left DLPFC rTMS was associated with increased left amygdala CBF [24]. In our study, rTMS to the DLPFC may be altering the efficiency of, or enhancing the “normal” regulation of the DLPFC on tonic amygdala activity, but the lateralisation was unexpected. In addition, no treatment-related changes in left amygdala CBF were observed, even at a liberal uncorrected threshold. Direct electrical stimulation of the right amygdala has been reported to elicit exclusively negative emotions, whereas left amygdala stimulation elicits both positive and negative emotional experiences [71]. Moreover, in healthy controls, harm avoidance traits have been more significantly associated with the strength of right amygdala resting-state connectivity with left hemispheric structures (including the left superior frontal gyrus [BA9] and basal ganglia) than was the left amygdala’s connectivity with those regions [72]. It may be that rTMS applied to the DLFPC in a clinical population characterised by high levels of avoidant behaviour, might modulate the resting activity of the contralateral amygdala.

Changes in amygdala CBF were not associated with changes in anxiety or depression. This is surprising given the central role of the amygdala in emotional processing and regulation. While the observed amygdala CBF changes were not associated with any shorter-term clinical changes, participants with the greatest rTMS-related reduction in amygdala regional CBF (i.e., between baseline and 1-month post-randomisation) showed the greatest sustained weight gain at 18-months post-randomisation. Research suggests hyper-activation of the amygdala is associated with fearful emotional processing of body images [73] and importantly, when at rest, elevated amygdala activity is associated with mental health disorders characterised by chronic anxiety (e.g., AN, generalised anxiety disorder, obsessive compulsive disorder). Therefore, it could be considered that a reduction in amygdala activity may be associated with an increased ability to tolerate uncomfortable physical and emotional sensations related to the body, which may in turn influence weight gain.

The above proposal is in accord with our data exploring whether baseline CBF could be used as a predictor of rTMS treatment response. Identifying such predictors may help to optimise treatment and identify appropriate patients for future studies [74]. We found that lower baseline CBF in the right amygdala was associated with a greater reduction in anxiety from baseline to 1-month post-randomisation, but this relationship did not persist. In contrast, higher baseline CBF in the insula predicted greater weight gain between baseline and 1-month post-randomisation and between baseline and 4-months post-randomisation. The insula has an essential role in monitoring body state, interoceptive awareness, and regulation of appetite and eating, and has been implicated in AN [75]. Furthermore, insula hyperactivity has been implicated in anxiety, and it has been proposed that self-starvation and the associated weight loss/low body weight may serve to regulate aversive emotions, such as anxiety, in AN (e.g., [76]), e.g., perhaps by blunting anxiogenic interoceptive and somatic signals processed in the insula. As early weight gain may predict longer-term treatment outcome in AN [77], it is possible that those with relatively preserved insula function are most likely to benefit from even a temporary TMS-induced blunting of the affective reactivity to interoceptive and emotional signals elicited by food perception or consumption. Alternatively, insula-mediated processes may directly underpin weight-gain following rTMS, but this seems less likely. Given the potential value of developing markers of treatment responses that could be assessed prior to investing in such a demanding rTMS treatment programmes, these preliminary findings warrant further investigation to establish the specificity of the association and clarify the underpinning mechanisms.

### Strengths and limitations

Our study is the first to explore rTMS effects on CBF in people with SE-AN, as part of a double-blind randomised controlled feasibility trial. ASL is a quantitative neuroimaging method with excellent/good test-retest reliability [78, 79]. In our analyses, we controlled for AN type and ED chronicity. However, a significant number of patients had to be discounted from the analyses due to incomplete datasets, rendering a relatively small patient group overall and particularly for the sham rTMS group and also for analyses on the predictors of long-term rTMS response using data from the 18-month post-randomisation follow-up. In addition, our sample only consisted of female participants but was heterogeneous across several other demographic and clinical parameters. As participants were community dwelling, we were unable to implement several factors that may be relevant for the reliability and clinical usefulness of our results e.g., standardised pre-scan meal, limit pre-scan exercise [80].

## Conclusions

In this exploratory study, we identified rTMS treatment related changes in amygdala CBF in adults with SE-AN. Participants receiving real rTMS showed greater reductions in amygdala CBF and this was associated with long-term weight gain. It may be that rTMS applied to the left DLFPC modulates the resting activity of the contralateral amygdala in a clinical population characterised by high levels of avoidant behaviour. Higher baseline CBF in the insula was also associated with greater weight gain between baseline and short-term follow-up and possible explanations are discussed above. Future rTMS investigations in AN should employ longitudinal neuroimaging to confirm and extend our findings.

## Supplementary Information

**Additional file 1: S1: Figure 1.** Participant flow through the trial with number of participants included in the cross-sectional and rTMS-related ASL analyses at each trial stage. **S2: Table 1.** Estimated Effect sizes (Cohen’s d), achieved power calculation and sample-size for future studies for neuroimaging contrasts estimated for independently derived anatomical regions of interest (ROIs) for significant results. **S3**: Association between illness duration and brain morphology. **Figure 2.** Regions where grey matter volume is positively associated with duration of illness.

## Data Availability

The datasets generated and/or analysed during the current study are not publicly available but are available from the corresponding author on reasonable request.

## Abbreviations

AC: Anterior commissure
AN: Anorexia nervosa
AN-BP: Anorexia nervosa binge-eating/purging type
AN-R: Anorexia nervosa restricting type
ASL: Arterial spin labelling
BMI: Body mass index
BN: Bulimia nervosa
CBF: Cerebral blood flow
CSF: Cerebrospinal fluid
DASS-21: Depression Anxiety and Stress Scales – Version 21
DLPFC: Dorsolateral prefrontal cortex
DMPFC: Dorsomedial prefrontal cortex
DSM: Diagnostic and Statistical Manual of Mental Disorders
ED: Eating disorder
EDE-Q: Eating Disorder Examination Questionnaire
fMRI: Functional magnetic resonance imaging
GM: Grey matter
HC: Healthy comparison
KCL: King’s College London
pCASL: Pseudo-continuous arterial spin labelling
PET: Positron emission topography
ROI: Region of interest
rTMS: Repetitive transcranial magnetic stimulation
SE-AN: Severe enduring anorexia nervosa
TI: Inversion time
TIV: Total intracranial volume
WM: White matter
