# Supplementary material for: A pilot study exploring the effect of repetitive transcranial magnetic stimulation (rTMS) treatment on cerebral blood flow and its relation to clinical outcomes in severe enduring anorexia nervosa
Source: J Eat Disord. 2021 Jul 9;9:84. doi: 10.1186/s40337-021-00420-w (PMC8268186; doi:10.1186/s40337-021-00420-w)
Supplement: Supplementary file 1 — Additional file 1: S1: Figure 1. Participant flow through the trial with number of participants included in the cross-sectional and rTMS-related ASL analyses at each trial stage. S2: Table 1. Estimated Effect sizes (Cohen’s d), achieved power calculation and sample-size for future studies for neuroimaging contrasts estimated for independently derived anatomical regions of interest (ROIs) for significant results. S3: Association between illness duration and brain morphology. Figure 2. Regions where grey matter volume is positively associated with duration of illness. [file 40337_2021_420_MOESM1_ESM.pdf]

A preliminary study exploring the effect of repetitive transcranial magnetic stimulation (rTMS) treatment on cerebral blood flow and its relation to clinical outcomes in severe enduring anorexia nervosa

Authors: Bethan Dalton, Erica Maloney, Samantha J. Rennalls, Savani Bartholdy, Maria Kekic, Jessica McClelland, Iain C. Campbell, Ulrike Schmidt, Owen G. O'Daly

Supporting Information

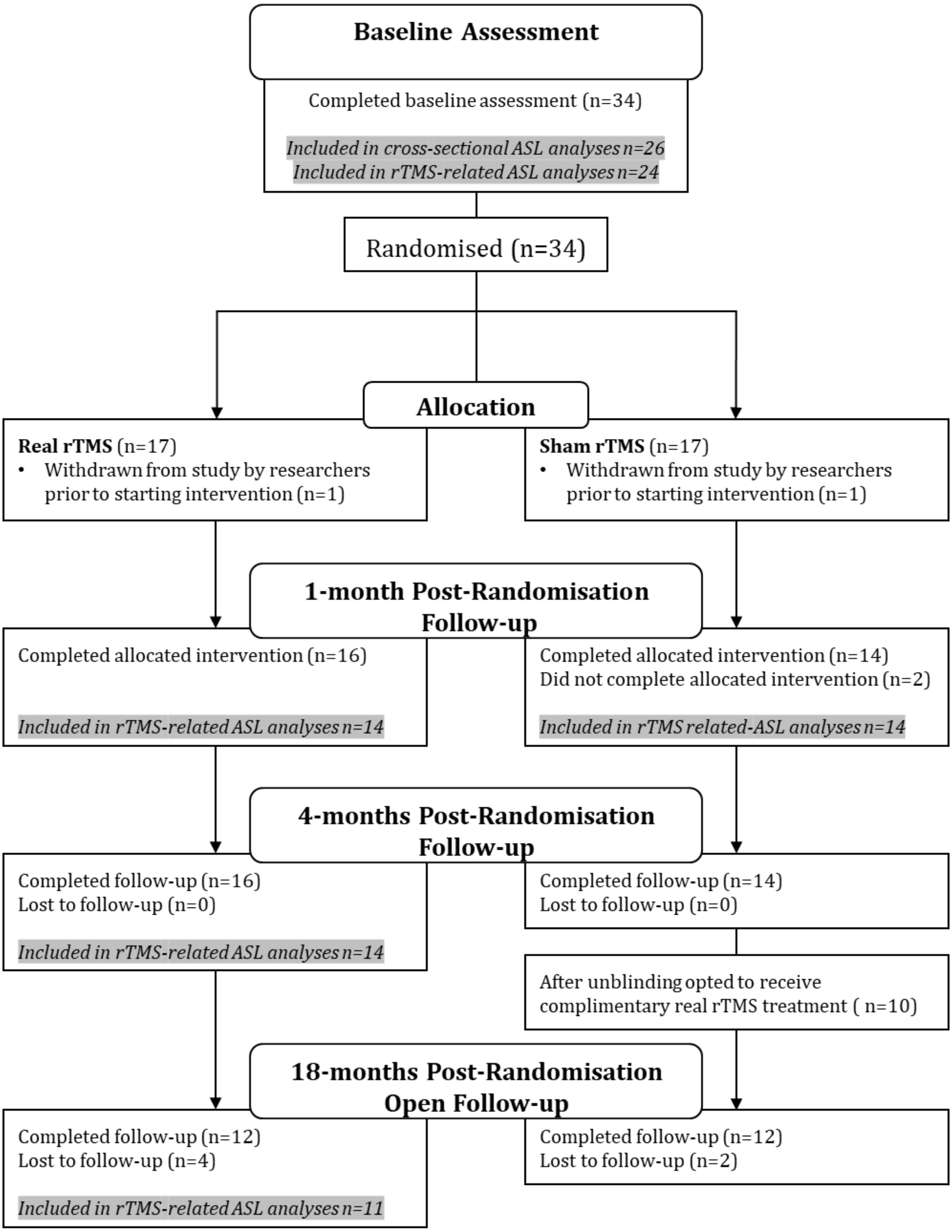

S1: Figure 1. Participant flow through the trial with number of participants included in the cross-sectional and rTMS-related ASL analyses at each trial stage.

S2: Table 1. Estimated Effect sizes (Cohen's d), achieved power calculation and sample-size for future studies for neuroimaging contrasts estimated for independently derived anatomical regions of interest (ROIs) for significant results.

Significant result	Effect size	Achieved Power	Required N
Two sample t-test			
Group difference in amygdala WM volume	0.24	0.14	216
Treatment x time interaction			
Amygdala CBF	0.32	0.19	122
Association between change in BMI over 18 months and change in CBF			
CBF in left insula	0.52	0.59	15
Right amygdala	0.65	0.67	13
Association between change in BMI over 1 month and baseline CBF			
Right insula CBF	0.39	0.69	18
Association between change in BMI over 4 months and baseline CBF			
Right insula CBF	0.25	0.52	27
Association between change in anxiety over 1 month and baseline CBF			
Left amygdala CBF	1.49	0.99	9
Right amygdala CBF	0.99	0.95	10
Association between illness duration and baseline GM volume			
Left insula GM volume	0.24	0.51	28
Thalamus GM volume	0.33	0.62	21

Abbreviations: WM = white matter; CBF = cerebral blood flow; BMI = body mass index; GM = grey matter.

S3: *Association between illness duration and brain morphology*

Due to the variance in illness duration in our SE-AN sample, we performed a whole-brain regression to test the association between grey matter volume and duration of illness, controlling for age, BMI and total intracranial volume. We found a significant positive association for the left insula (pFWE = 0.019, Z = 4.27, [-26 22 -13]; see Figure 2), but not the amygdala or anterior cingulate cortex. These hypothesis-led analyses used bilateral regions of interest (ROI) masks for these three structures. A subsequent exploratory whole-brain analysis in the thalamus showed a significant association with illness duration (pFWE= 0.009, cluster-size = 503 voxels, [3 -8 6]; see Figure 2).

We then performed a whole-brain regression to test the association between white matter volume and duration of illness, controlling for age, BMI and total intracranial volume, finding no associations between white matter volume and duration of illness, both for our hypothesis-led ROIs and an exploratory whole-brain analyses.

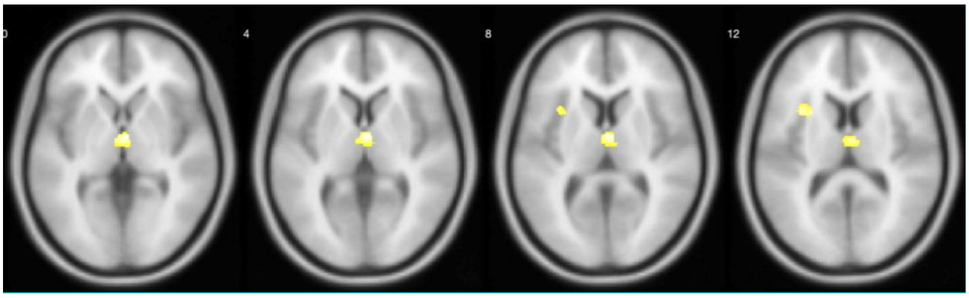

Figure 2. Regions where grey matter volume is positively associated with duration of illness.
